# Candidiasis in breast cancer: Tumor progression or not?

**DOI:** 10.22038/ijbms.2024.75408.16379

**Published:** 2024

**Authors:** Somayeh Ashrafi, Abbas Ali Amini, Pegah Karimi, Maryam Bagherian, Mohammad Mehdi Adibzadeh Sereshgi, Fatemeh Asgarhalvaei, Khadijeh Ahmadi, Mohammad Hossein Yazdi, Hamid Reza Jahantigh, Mehdi Mahdavi, Ramin Sarrami Forooshani

**Affiliations:** 1 Advanced Therapy Medicinal Product (ATMP) Department, Breast Cancer Research Center, Motamed Cancer Institute, Academic Center for Education, Culture and Research (ACECR), Tehran, Iran; 2 Recombinant Vaccine Research Center, Faculty of Pharmacy, Tehran University of Medical Sciences, Tehran, Iran; 3 Department of Microbiology, Faculty of Advanced Sciences & Technology, Tehran Medical Sciences, Islamic Azad University, (IAUPS), Tehran, Iran; 4 Department of Immunology, Faculty of Medicine, Kurdistan University of Medical Sciences, Sanandaj, Iran; 5 Cancer and Immunology Research Center, Research Institute for Health Development, Kurdistan University of Medical Sciences, Sanandaj, Iran; 6 Department of Biochemistry, Faculty of Basic Sciences, Islamic Azad University, Central Tehran Branch, Tehran, Iran; 7 Department of Hematology and Oncology and Stem Cell Transplantation, Firoozgar Hospital, School of Medicine, Iran University of Medical Sciences, Tehran, Iran; 8 Department of Immunology, Shahed University, Tehran, Iran; 9 Infectious and Tropical Diseases Research Center, Hormozgan Health Institute, Hormozgan University of Medical Sciences, Bandar Abbas, Iran; 10 Biotechnology Research Center, Tehran University of Medical Sciences, Tehran, Iran; 11 Immunotherapy Group, Pharmaceutical Sciences Research Center (PSRC), The Institute of Pharmaceutical Sciences (TIPS), Tehran University of Medical Sciences, Tehran, Iran; 12 Animal Health and Zoonosis PhD Course, Department of Veterinary Medicine, University of Bari, Bari, Italy; # These authors contributed eqully to this work

**Keywords:** Breast cancer, Cancer therapy, Candida albicans, Immune responses, Tumor progression

## Abstract

*Candida albicans* is an “opportunistic fungal agent” in cancer patients that can become colonized in both mucosal and deep tissues and cause severe infections. Most evidence has shown that *C. albicans *can enhance the progress of different cancers by several mechanisms such as generating virulence factors, participation in endogenous production of pro-inflammatory mediators, and stimulating a wide range of immune cells in the host. The main idea of this review is to describe a range of *Candida*-used mechanisms that are important in candidiasis-associated malignant processes and cancer development, particularly breast cancer. This review intends to provide a detailed discussion on different regulatory mechanisms of *C. albicans *that undoubtedly help to open new therapeutic horizons of cancer therapy in patients with fungal infection. The current therapeutic approach is not fully effective in immunocompromised and cancer patients, and further studies are required to find new products with effective antifungal properties and minimal side effects to increase the susceptibility of opportunistic fungal infections to conventional antifungal agents. So, in this situation, a special therapy should be considered to control the infection and simultaneously have the most therapeutic index on tumor patients.

## Introduction

Breast cancer is the most common cancer and one of the leading causes of death among females worldwide (1). Therapeutic interventions like chemotherapy, targeted therapy, and immunotherapy may lead to alterations of the immune system rendering these patients susceptible to infectious complications. Studies have demonstrated that most women with breast cancer exhibit a high prevalence of opportunistic fungal infection, especially *Candida albicans* (2). 

*C. albicans* is a commensal organism living with humans responsible for opportunistic infections in immunodeficient patients (3). Systemic *C. albicans* infections may negatively affect the outcome of malignancy and produce lengthy hospital stays, intensify the economic burden of disease, and increase morbidity and mortality (4). In patients with a moderately compromised immune system, *C. albicans* often induce mild or superficial infections, however, this fungus may also establish life-threatening diseases (5). 

The role of microorganisms in cancer incidence and morbidity along with their interactions with the immune system and host responses have been explored. However, the impact of fungi on carcinogenesis is not clearly understood mainly because of their much lower prevalence and cumbersome investigation techniques. Interrelation of *C. albicans* to cancer development like colorectal carcinogenesis has been shown (6-8). The primary purpose of this review is to explore the effects of candidiasis on the immune system and tumor cells and highlight some recent findings suggesting that *C. albicans* may have a more extensive role in breast cancer status or progression. 

***The host immune response against Candida albicans***

Immunological crosstalk between the host immune system and *C. albicans* is complex and dynamic since the pathogen employs various strategies to escape antimicrobial immunity (9, 10). Upon initial fungal infection, both innate and adaptive immune reactions restrict fungal proliferation through various approaches. 

***Innate immune response against C. albicans***

*Candida* expresses many pathogen/microbe-associated molecular patterns (PAMPs/MAMPs), including N-linked mannan, β-glucan, α-mannans, acylated lipoprotein, and β-(1-2) oligomannan, which are involved in anti-fungal immunity (11). The interplay between PAMPs and pattern recognition receptors (PRRs) determines the path of inflammation or infection. PRRs activation is accompanied by activation of phagocytosis, transcriptional factors, nuclear factor kappa-light-chain-enhancer of activated B cells (NF-κB), activator protein 1 (AP-1), interferon regulatory factors (IRFs), CCAAT/enhancer-binding protein beta (C/EBPβ), proinflammatory cytokines production, and inflammasome activation which trigger inflammatory pathways (12, 13). 

The primary cells that are involved in innate immunity against *Candida* are epithelial and phagocytic cells, including polymorphonuclear neutrophils (PMNs), mononuclear phagocytes, monocytes/macrophages, dendritic cells (DCs), and natural killer cells (NKs) (11). Among them, epithelial cells of the skin, urogenital, gastrointestinal, and respiratory systems are the first line of host defense. The fungus exploits two ways to attack the human host tissues and spread to all other regions of the human body: passive penetration (endocytosis) and active penetration (14). Passive penetration is mediated through the interaction of several adhesions of *C. albicans* including hyphal wall protein1 (HWP1), agglutinin-like sequence 1-9 (ALS1-9), and integrin-like protein 1 (INT1) (15). In contrast, active penetration is directly dependent on fungus features including touch (thigmotropism), hyphal-induced physical pressure, and the secretion of extracellular hydrolases like Saps, class B phospholipase (Plb), and lipase (Lip) families (16, 17).

The interplay between *C. albicans* and epithelial surfaces encourages signaling pathways like NF-κB and biphasic Mitogen-activated protein kinase (MAPK). It has been shown that activation of NF-κB, first MAPK phase, ERK1/2, and JNK signaling further promote the expression of antimicrobial peptides like defensins, cathelicidins, and statins (18, 19). The activation of the second MAPK phase is dependent on the hyphal form of *C. albicans* and is associated with inducing c-Fos activity that triggers the secretion of pro-inflammatory molecules such as IL-1α/β, IL-6, IL-8, Tumor necrosis factor alpha (TNF-α), Granulocyte-macrophage colony-stimulating factor (GM-CSF), and Granulocyte colony-stimulating factor (G-CSF) in vulvovaginal candidiasis (20, 21).

Furthermore, the secretion of some pro-inflammatory mediators such as IL-22 leads to immune cell proliferation, differentiation, and activation (22). The overexpression of IL-22 together with other mediators such as TNF-α and IL-17 stimulates the production of the complement system components C1r, C1s, and anti-fungal peptides by epithelial cells (23, 24). Depending on the type of infection, epithelial cells may promote and generate chemokines to call up neutrophils towards infectious niches to directly kill *Candida* cells (25). These cells recruit several anti-fungal mechanisms to demolish *Candida* cells including phagocytosis, cytokine secretion, granule enzymes production, antimicrobial peptides, and oxidative burst; latent of which results in the generation of reactive oxygen species (ROS), nitrogen intermediates, and myeloperoxidase (MPO) (26, 27). 

The infiltration of neutrophils inhibits *C. albicans* growth and promotes the yeast-to-hyphal transition (9). Urban *et al*. showed that neutrophils are equipped with neutrophil extracellular traps (NETs), which can destroy both yeast and hyphal forms of the fungus, enabling extracellular killing of the microorganism. (28). The bactericidal/permeability-increasing (BPI) protein, lactoferrin, and defensins are among the anti-fungal proteins found in NET granules and are involved in pathogen killing (25). 

Monocytes and macrophages are other phagocytic cells directly involved in anti-*Candida *immune responses (29, 30). Macrophages utilize a combination of several oxidative and non-oxidative anti-fungal mechanisms including phagocytosis, antimicrobial peptides, degradative enzymes inducing ROS and nitric oxide synthase (iNOS), and formation of macrophage extracellular traps (METs) to attack the invasive pathogens (31, 32). As professional antigen-presenting cells (APCs). DCs also play an essential role in regulating immune responses and bridging the innate to adaptive anti-fungal immune trajectories (33). 

DCs possess several PRRs enabling them to localize infection and activate naïve T cells (34). Furthermore, cytokines secreted by DCs lead T cells to differentiate into Th1, Th2, Th17, and Tregs. In response to the yeast form of the pathogen, DCs are activated and secrete IL-12 which promotes Th1 cells, while ingestion of hypha form triggers IL-4 production and Th2 differentiation (35). Interestingly, Dectin-1 interaction with the yeast form induces IL-17 and IL-6 overexpression promoting Th17 cell responses that protect cutaneous infection. Filamentous form, in contrast, provokes just Th1 differentiation, which is essential for the control of systemic infection (36). 

Additionally, the cell wall protein fraction (CPF) of *C. albicans* initiates MHC-II, CD86, and CD40 expression on dendritic cells, which indicates DCs maturation (37). Van de Veerdonk studied the innate immune mechanisms involved in triggering Th17 responses and showed that *C. albicans* mannan, macrophage mannose receptor (MR), and TLR2/dectin-1 pathway activate and induce IL-17 production as a pathogen-specific defense (38). Moreover, it is shown that vaccination with recombinant cell surface glycoprotein Als3p, a significant component of the hyphal form, induces Th1, Th17, and Th1/17 lymphocytes, resulting in decreased tissue infectious burden (39). 

NK cells are innate cytotoxic lymphocytes that can directly and potentially recognize and phagocytize *C. albicans *cells by releasing molecular contents such as perforin and granzymes, causing receptor-mediated apoptosis (40, 41). They usually have effective roles in anti-viral and anti-tumor immunity as well (42). These cells modulate various innate and adaptive immune cells and responses through secretion of various pro-inflammatory mediators (43). A study showed that NK cells in defense against *C. albicans* infection exhibit different roles depending on the state of host defense and immunological context. This study demonstrates that NK cells cause hyper/chronic inflammation in candidiasis and immunocompetent hosts by stimulating excessive pro-inflammatory mediators, which may be redundant and even detrimental to host defense and results in the exacerbation of infection (44). 

***Adaptive immune response against C. albicans*** 

Adaptive immune responses are mainly carried out by two cell types: T lymphocytes and B lymphocytes. T lymphocytes are divided into CD8+ cytotoxic cells and CD4+ helper (Th) cells, both of which participate in anti-fungal immunity and their activation is monitored by DC subsets that migrate to the local lymph nodes. Activation of different subtypes of DCs via distinct signaling pathways can shape diverse T-cell responses against *Candida *infections (45, 46). The importance of CD4+ helper T cells is well recognized in host defense against *Candida *infections (47). Th1, Th2, Th17, and Tregs, all play pivotal roles in *Candida*–specific immune reactions. 

Th2 and Th17 mediated reactions are initiated by collaboration of myeloid (inflammatory) dendritic cells via TLR-MyD88 pathways, whereas Th1 and T regulatory (Treg) cell responses originate in plasmacytoid (tolerogenic) dendritic cell interaction via TRIF signaling pathways (48). Several inflammatory cytokines including IFN-γ, IL-12, IL-1β, IL-2, IL-6, TNF-α, IL-17, IL-21, IL-22, and IL-23 are bound to Th1 and Th17 cell mediated immunity (46). In a 2007 research, it was demonstrated that IFN-γ knockout mice show significantly lower surveillance while surveillance rate was increased in IL-4, a Th2 cytokine, knockout mice (49). This finding indicates a protective role of Th1 and detrimental effect of Th2 response, which confirms the importance of Th1/Th2 balance against *Candida* infections (48, 50). 

Notably, another study represented that as with epithelial cell responses, various Th phenotypes-specific responses against *Candida* infections are tissue-specific (51). Recognition of *Candida* by PRRs on DCs such as dectin-1 and dectin-2 promotes Th17 proliferation and development (52). On the other hand, Th17 and its related cytokines, IL-17 and IL-23, play a crucial role in protective immunity and reduce fungal burdens in vaginal candidiasis (53). Additionally, Th1 and Th17associated cytokines, IFN-γ and IL-17, can prohibit cancer development, and as a result, suppressing these cytokines may increase the risk of cancer in the candidiasis tissue environment (54). 

Considering the importance of neutrophils for anti-fungal immunity, IL-17R deficiency could indirectly affect these cells by impairing NK cell functions. Simultaneously, GM-CSF not only induces neutrophil migration and *Candida* killing but also stimulates oxidative metabolism (55-57). In addition, defects in GM-CSF signaling increase the risk of invasive aspergillosis infection, and aligned with this finding, GM-CSF treatment could exacerbate the fungicidal activity of neutrophils and monocytes and improve fungal clearance in the lung (58). 

Treg cells are a subpopulation of CD4+ helper T cells, which are involved in controlling inflammation, autoimmunity, and hemostasis (59, 60). Tregs can potentially exhibit opposite features during infections and breast cancer. For instance, they may facilitate tumor growth and metastasis by suppressing most immune cells including CD4+ and CD8+ T cells, B cells, NK cells, and APCs; or enhance microbial clearance and cancer improvement through stimulation of immune mechanisms (61). Despite the immune-suppressing function of Tregs, their role in combatting *Candida* is controversial. In a report of oral *Candida* infection in mice, Treg cells enhanced Th17 differentiation by inducing IL-17 secretion and IL-2 consumption, which helped fungal clearance (59). However, in another study on mouse models, Treg cell depletion or IL-10 administration did not result in Th17 cell response to *Candida* in the murine oral epithelium infection (62). Nonetheless, the intravenous injection of infection-induced Foxp3+ Treg cells in C57BL/6 mice was associated with fungal kidney infection (63). In line with previous findings, this study indicated the role of Tregs in Th17 responses but showed suppressive effects of these regulatory cells in the down-regulation of Th1 and Th2 pathways (59, 62). Also, our previous finding showed that *C. albicans* could induce Treg homing in the tumor microenvironment, which led to increased tumor growth (64).

**Figure 1:**
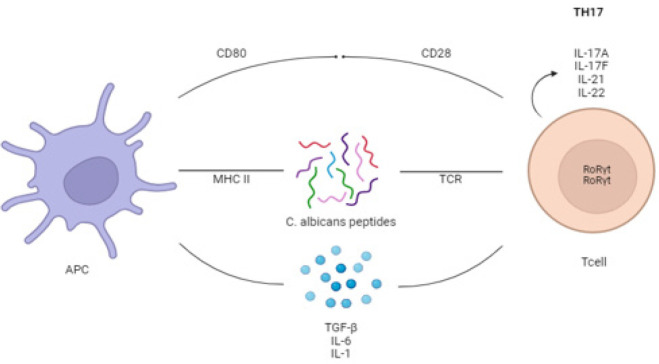
The effect of *Candida albicans* products on the modulation of T cells and induction of suppressor cytokines

CD28: Cluster of Differentiation 28; MHC-II: Major histocompatibility complex-II; TCR: T-cell receptor; TGF-β: Transforming growth factor beta; APC: Antigen-presenting cell; ROR-γt: Retinoic acid receptor-γt

**Figure 2:**
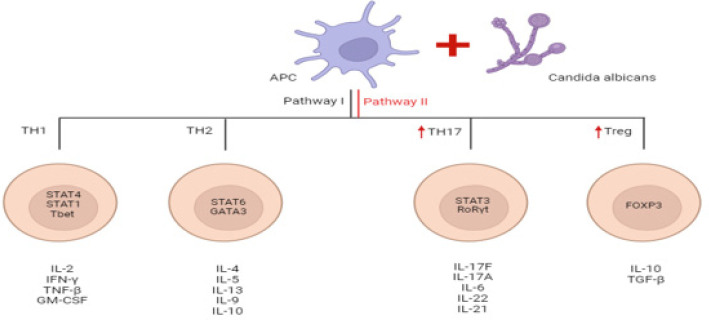
Interaction of *Candida albicans*and antigen presenting cells can modulate T cells response and cytokines pattern

TH: T helper; Treg: Regulatory T cell; STAT: Signal transducer and activator of transcription proteins; Tbet: T-box transcription factor; IFN-γ: Interferon gamma-γ; TNF-α: Tumor necrosis factor-α; GM-CSF: Granulocyte-macrophage colony-stimulating factor; GATA-3: GATA binding protein 3; FOXP3: Forkhead box protein 3

Collectively, innate and adaptive immune responses consist of a wide range of molecules and receptors that communicate with each other in a coordinated manner to provide host protection against pathogens. However, our knowledge of immune responses against fungal pathogens is inadequate and incomplete, and possibly further research would help us better understand how immune signals interact and how invading fungi hide themselves from immune cells and escape from the immune system to develop cancer-related processes (Figure 1 and 2).

**Role of** ***C.*** ***albicans* in tumorigenesis **

A large body of evidence exists that fungi participate in the processes that encourage carcinogenesis and cancer progression (65, 66). There are several pathways through which, *Candida* spp. increase the risk of cancer and metastasis (67), namely the generation of carcinogenic products (nitrosamine, acetaldehyde), inducing inflammation (inflammatory mediators, cytokines, and chemokines), molecular mimicry, and epigenetic modifications.

***Generation of carcinogenic by-products***

*Nitrosamine*

N-nitroso compounds or nitrosamines are chemical carcinogens of nitrogen oxides (e.g., nitrites and nitrates). Nitrosamines react with DNA and form adducts with phosphate residues that may stimulate the activation of specific proto-oncogenes (68, 69). There is much evidence linking nitrosamines to the incidence of various types of cancer, including colorectal, stomach, esophagus, nasopharynx, bladder, and breast (70-73). Studies have shown that 4-(methylnitrosamino)-1-(3-pyridyl)-1-butanone (NNK), the most potent carcinogen among tobacco-related nitrosamines, can affect various neoplastic processes and increase the risk of developing breast cancer by stimulation of estrogen (73). 

Moreover, a study reported that some yeasts like *C. albicans* might have a role in oral cancers through endogenous nitrosamine breakdown (74). Invasive esophageal *Candida* infection is also associated with dysplastic alterations in the oral epithelium and esophageal squamous cell carcinoma (OSCCs) (75-77). Furthermore, among different grades of leukoplakia, *C. albicans* growth was observed only in severe dysplastic patients, while there was no growth in mild or moderate cases. The evidence demonstrated that *C. albicans* production of nitrosamines might indirectly promote cancer progression (78). In addition, the expression of wild-type BRCA1 suppresses the growth of breast and ovarian epithelial tumor cell lines (79). The study by Humphrey *et al*. showed that BRCA1 inhibited yeast growth by several mechanisms, and mutations in this gene may promote breast cancer progression and yeast dissemination (80). 

Furthermore, previous reports showed that the level of active-matrix metalloproteinases (MMPs), especially MMP-2, could be considered a breast cancer metastasis indicator. MMPs are secreted as pro-enzymes, activated by proteolytic cleavage, and regulated by a family of inhibitors (i.e., tissue inhibitors of matrix metalloproteinases; TIMPs). Previous studies showed that TIMP-1 expression is enhanced in breast cancer and possibly other types of cancer. In this regard, Taheri *et al*. showed that *C. albicans* stimulates MMP-9 secretion in mice-bearing tumors and seems to utilize MMP-9 to degrade the tissue and disseminate. Also, they demonstrated that TIMP-1 was increased in the presence of infection and tumor. Overall, the results showed that candidiasis positively affected tumor progression and metastasis. (4, 81). It can be concluded that *Candida*, by alteration of tumor suppressor genes and proto-oncogenes, provides a context for tumor initiation and progression.

*Alcohol-derived carcinogenic agents*

Alongside nitrosamines, *Candida* takes advantage of another pathway to promote carcinogenesis, especially for oral carcinoma. *C. albicans* utilizes the enzyme alcohol dehydrogenase (ADH1) to oxidize ethanol of alcoholic beverages and some substances such as carbohydrates, producing elevated levels of acetaldehyde (ACH) (>100 µM), which is highly toxic, mutagenic, and carcinogenic; thus indisputably elevates the risk of carcinoma (82). Binding to proteins and DNA, acetaldehyde creates abnormal chromosomal aberrations, alters molecules’ typical structure and function, and induces inflammation by producing inflammatory mediators such as NF-κB in the trachea (83). Moreover, acetaldehyde triggers mitochondrial damage and boosts ROS (84). These established alterations cause genome instability, suppression of the apoptotic process, proto-oncogene activation, and cell cycle disturbances, which may favor tumor progression (85). 

*C. albicans* could also induce oral epithelial dysplasia (86). In an *in vitro* study, L-2-hydroxyisocaproic acid (HICA), a novel antifungal agent, completely inhibited ACH production, reducing the mutagenic potential of *C. albicans* biofilms (87). In addition, a study represented that intracellular ethanol metabolism to acetaldehyde causes DNA damage, causing Fanconi anemia-breast cancer (FA-BRCA) susceptibility in alcohol-associated breast and liver cancers (88). Overall, it seems that *Candida*, via production of alcohol-derived carcinogenic agents, can stimulate breast cancer.

*Heme oxygenase (HO) enzymes*

Hemoglobin (Hb) is considered an iron-containing oxygen-carrier metalloprotein (89). Heme oxygenase enzymes are found in bacteria, fungi, and mammals. *C. albicans* heme oxygenase gene (CaHMX1) exploits extracellular heme or Hb as a significant source of iron to degrade the ferroheme to free iron, biliverdin, and carbon monoxide (CO) and allow the fungus to feed on the iron to support microbial growth and pathogenesis (90, 91). Moreover, when *Candida *faces iron deprivation, hemin induces the Hmx1 gene, sustaining fungal survival and virulence (91). 

In addition to hemolytic capacity, Rbt5 and Pga7, two extracellular membrane proteins, help the yeast to transfer iron from heme and hemoglobin (92, 93). Besides these two proteins, in the hyphal form, another member of the Rbt5 protein family, Csa2, is also involved in iron absorption (94). In 1966, it was shown that endogenous CO production is correlated with blood heme destruction (95). This study showed that CO, despite its poisonous effects, could be considered an immune modulator at therapeutic doses. Up-regulating Hmx1, *Candida* can alter the immune system. Like human heme oxygenase, *Candida* Hmx1 could produce CO, which leads the immune system toward Th2 expression by diminishing cellular Th1 immunity and weakening antigen presentation. It must be considered that Th2 cells facilitate tumor progression. Evidence shows that Th2 predominance enables tumor growth in pancreatic cancer, and a higher Th2/Th1 lymphocyte ratio could affect the survival rate after surgery (95). 

Our previous findings demonstrated that *C. albicans* infection was followed by decreased IFN-γ/IL-4 ratio and increased IL-10 and TGF-β, which results in augmented tumor growth (95). Although it is expected that Hmx1, by shifting immune responses to Th2, might facilitate tumor growth, evidence showed the beneficial role of HO-1 in blocking breast tumor invasion (96, 97). Other investigations also represented HO-1 inhibiting effect on the TPA-induced MMP-9 expression and invasiveness with activation of PKC/ROS/extracellular signal-regulated kinases (ERK) cascade in the human breast carcinoma cells (97). Taken together, *C. albicans* can contribute to cancer progression by producing HO-1 and its derivatives, but this factor may not be its only promoter for cancer development.

***Inflammatory response***

Interactions between specific PAMPs and PRRs, such as TLR2, TLR4, dectin-2, dectin-1, etc., on the surface of epithelial and myeloid cells, activate the inflammatory cascades by triggering the expression and secretion of a broad range of molecules including cytokines, cell growth factors, cell adhesion molecules, and immune receptors (98, 99). In immunocompromised cancer patients with a reduced number of leukocytes and other inflammatory mediators, circulating tumor cells can adhere and attach to the endothelium instead of leukocytes, which could potentially be the first step in creating secondary tumors and metastasis (67). 

Inflammatory response of endothelial cells mediates high tumor cell adhesion and metastasis after being stimulated by *C. albicans*. *Candida* recognition by PRRs leads to stimulation and activation of multiple intracellular signaling pathways including Nuclear factor of activated T-cells (NFAT), NF-κB, Mitogen-activated protein kinases (MAPK), and extracellular-signal-regulated kinase (ERK), which result in the secretion of several cytokines like IL-1, IL-2, TNF-α, IL-8, IL-6, IL-10, and IL-12 (11). It is reported that *Candida* infection would interrupt the integrity of gut mucosa in the intestinal epithelial cells (IEC) model (100). Moreover, many findings indicate the prominent role of the MAPK and NF-κB pathways in inducing proinflammatory milieu in *Candida* infection. The extracellular signal-regulated kinase (Erk) MAPK pathway prevents apoptosis of CD8+ T cells by modulating the expression of Bcl-2-interacting mediator of cell death (BIM), B-cell lymphoma 2 (BCL-2), and B-cell lymphoma-extra-large (Bcl-XL) proteins (101). MKPs play an essential role in innate immune responses by negatively regulating MAPK. MKP-1^-/-^ mice produce hefty amounts of TNF-α, IL-10, IL-1β, and IL-6 when challenged with LPS, making them hyper-responsive to endotoxin shock (102). 

Furthermore, the NF-κB pathway is another important route that exerts acute effects on the development and function of the immune system (102, 103). For instance, Gratacep *et al*. showed that high-level mucosal infection with *Candida* induces NF-κB activity while low-level infection does not affect this pathway in NF-κB activity (104). Also, this study demonstrated that only direct contact of the yeast to epithelial cells could induce NF-κB (105, 106). 

Numerous studies reported that *C. albicans* affect cancer progression and metastasis through proinflammatory pathways in a cytokines-dependent manner and expression of adhesion molecules (107). In this regard, in a previous study, we have shown that *C. albicans* infection in the tumor-bearing mice made a dysregulation in cytokine profiles and could facilitate tumor growth and skewed immune responses toward Th2 in the breast tumor microenvironment (64). Also, several experiments have shown that systemic candidiasis is accompanied by augmentation of anti-candida Th1-related responses that promote the secretion of various cytokines like IFN-γ, TNF-α, TGF-β, IL-6, IL-12, IL-15, and IL2. In preclinical studies, proinflammatory cytokines, such as IL-12, IL-15, and TNF-α, exhibited adjuvant activity because they could up-regulate the protective anti-fungal Th1 response and block Th2 immune reaction (108). 

On the other hand, an elevated Th17-induced inflammatory response may increase pathogenicity correlated with *C. albicans* survival and dissemination in the mouse model and impair protective immunity (109). Among the proinflammatory cytokines, TNF-α, IL-1β, IL-6, IL-8, and colony-stimulating factors (CSFs) are essential cytokines involved in the host-*Candida* interactive communication. Although IL-1β and IL-6 play an essential role in PMN infiltration, they are not as crucial as TNF-α in the anti-fungal innate response. Interestingly, previous infection with *C. albicans* can mediate cancer initiation and progression by increasing the final level of TNF-α and IL-18 (107). 

Additionally, some investigations have suggested that TGFβ restricts the phagocytic capacity of activated monocytes-macrophages in *C. albicans* infection-bearing mice and leads to inhibition of IFN-γ-induced nitric oxide production, which may facilitate the progression of *C. albicans* infection (110). However, TGF-β plays dual roles in tumor environment and normal cells. Researchers argue that during the initial stages of tumor outgrowth, TGFβ acts as a tumor suppressor, preventing its progression to malignancy (111). However, during late-stage human breast tumors, TGF-β expression increases, which can exert angiogenic and immunosuppressive effects in the tumor microenvironment facilitating tumor progression (111, 112). Thus, invasive *Candida* infection in the chronic stages can significantly promote the spread of cancer by stimulating the secretion of TGF-β as an immunosuppressive agent. 

In addition to the previously described mechanisms, evidence shows the dominant function of CD4+ T-cell subsets called Th17 cells, in response to *C. albicans *(113). Th17 cells are a fascinating subset that play significant roles in inflammatory diseases and protection against opportunistic pathogens and cancer (114). However, the role of IL-17 in candidiasis is controversial. Some mouse studies showed a potential association of IL-17 and IL-23 with candidiasis, but others have failed to find a strong connection (115). Most probably, IL-17 plays tissue-specific roles in immunity to *C. albicans*. Anti-*Candida* activity of IL-17 was first shown in 2004. Although Th17 is a directly essential cytokine against *Candida* infection, other Th17-related cytokines such as IL-23 showed tumorigenic and metastatic properties and influenced the pathogenic potential of Th17 cells in neoplastic microenvironments (116). Also, cancer progression is indirectly promoted by IL-17 via recruiting phagocytes, particularly neutrophils (117).

***Molecular mimicry***

The adhesion profile is considered the first step in initial fungal infection that leads to colonization, free dissemination, and invasive infections (118). Numerous investigations indicated that some *C. albicans* surface proteins such as complement receptor 3-related protein (CR3-RP), have structural and antigenic homology with glycoprotein CD1lb/CD18 on leukocytes, which are essential agents for adhesion of leukocytes to the endothelial cells (119, 120). CD1lb/CD18 is found in human neutrophils, monocytes, and macrophages. Polyclonal or monoclonal antibodies that recognize a subunit of CD1lb/CD18 and target CR3-RP of *C. albicans* may crosstalk with CR3 of leukocytes and impair host anti-*Candida* and anti-tumor immunity. This is called molecular mimicry, favoring invasive *Candida* infection and cancer progression (121).

***Epigenetic modification***

Existing data suggest that pathogenic fungi can create diversity and genome plasticity in response to stressful growth conditions by chromosomal variation and increasing copy number (122, 123). Among the *Candida* species, *C. albicans* displays extensive genomic diversity and plasticity in the *de novo* format (123, 124). Asexual mitotic genome rearrangements have been identified as the central genomic diversity in *C. albicans* variants (125). Diverse repetitive loci of DNA are commonly compressed at ribosomal DNA (rDNA) sites, centromeres, and telomeres and are assembled into heterochromatin structures and organize long repetitive sequences that contribute to genotypic phenotypic plasticity (126, 127). There are at least four major groups of long repetitive sequences in *C. albicans*: tandem telomeric repeat, long/major tandem sequences (MRS), long terminal repeats (LTRs), and ribosomal DNA repeats (rDNA) (128-130). These long repetitive sequences experience recombination in both inter- and intra-genic occurrences that immediately generate long chromosomal polymorphisms, chimeric chromosomes, and telomere-telomere chromosomal fusions (131, 132). 

Heterochromatic regions exert a transcriptionally repressive environment that can disseminate over long distances (up to 50kb), occasionally silencing native genes such as reporter genes inserted at these regions independently of the essential DNA sequence (133, 134). Histone modifiers can manage the transcriptionally repressive level of heterochromatin regions through chromatin modifications. In this regard, Sitterlé *et al*. evaluated the chromatin states correlated with DNA repeats in *C. albicans*. Their results indicated that in this species, differential heterochromatin states regulate gene expression independently of the DNA sequence, and heterochromatin remodeling is associated with adaptation in a stress situation (135). Furthermore, numerous studies have reported that some non-coding RNAs including microRNAs, have a noticeable capacity to regulate proto-oncogenes such as PIM-1 (136). Since the expression of miRNAs occurs in tissues and a tumor-specific manner, it seems that some miRNAs are subject to epigenetic regulation. 

**Figure 3:**
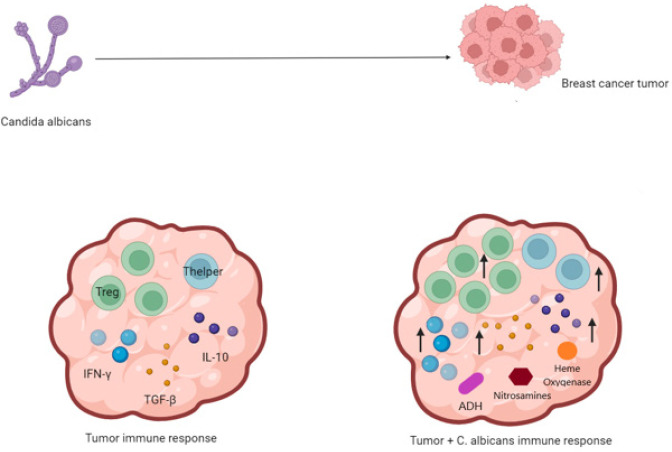
*Candida albicans *can modulate tumor microenvironment through secretion of different molecules

Circular RNAs (circRNAs) generated during the alternative RNA splicing process could promote breast cancer cell progression under hypoxia (137). In addition, some studies have indicated that circRNAs might also be involved in breast cancer proliferation and migration (138). On the other hand, defective matches can be established in circRNA-miRNA duplex, enabling circRNAs to serve as “miRNA sponges” and suppress miRNA-mediated degradation of mRNAs (139). Gene ontology (GO) enrichment analysis indicates that invasive *Candida* may influence the regulation of respiratory epithelial functions by interference in different miRNA expressions and alteration of many critical biological pathways (140). Also, the results of one study showed that heat-killed *C. albicans*, accompanied by other factors triggering the NF-κB and anti-inflammatory cytokines, could induce/inhibit specific miRNAs and regulate functions of innate immune cells such as macrophages following PRR stimulation (141) (Figure 3).

**Therapeutic approaches**

Due to the high compatibility and flexibility of *C.* *albicans* to grow in different host niches, it has been identified as an important and prevalent species in cancer incidence and development (142). Now, many fungicidal agents/drugs have been developed to treat systemic candidiasis infection. The most common structural classes of anti-fungal agents include polyenes (the oldest class of anti-fungal drugs), fluoropyrimidines, echinocandins, and azoles. Each of them constitutes several subclasses that pursue different pathways to eradicate fungal-related infections (143). The innovation of new alternative compounds, alone or in combination with other anti-fungal agents can increase anti-fungal capacity (144). In this regard, Onyewu and Heitman reported that the combination of 4T1 cell lysates and heated *C. albicans* extract, by induction of both innate and adaptive immunity, amplified anti-tumor immune responses, enhanced survival rates, and reduced tumor volume in the murine model of breast cancer (143, 144). 

Comprehensive and exploratory studies evaluated the anti-morphogenetic properties of thirty anti-cancer agents on the yeast to hyphal form transition of *C. albicans* and provided the possibility of repurposing and designing cancer drugs as anti-morphogenetic agents in cancer-related *C. albican*s infection (145, 146). A research study indicated estrogen antagonist tamoxifen suppressed the growth and dissemination of fluconazole (FCZ)- sensitive *C. albicans* isolated from periodontal patients (147). However, tamoxifen’s exact mechanism of action as an anti-fungal agent is unknown. The drug may be involved in preventing pathogenic fungal growth and development by induction of calcium-calcineurin signaling pathway and blocking calmodulin signaling (148, 149). 

Many fungal pathogens evolved anti-oxidative factors to mediate survival during infection. But, in hypoxic and anoxic circumstances, neutrophil’s natural function to efficiently generate reactive oxygen species is impaired. To this end, studies have shown that disrupting fungal redox balance could be a new therapeutic approach for producing effective and suitable drugs. Tempol, a redox-cycling nitroxide, is an anti-cancer and anti-inflammatory drug that has recently been proven to have anti-fungal effects, especially in response to systemic *C. albicans* (150). Besides, the repurposing of traditional compounds/drugs for new targets can shorten the treatment time and provide rapid therapy and novel opportunities to develop de novo anti-fungal agents (151). 

Although the demand and consumption of fungicidal drugs in modern healthcare have increased significantly over the last decades, the rate of available therapeutics may not meet these demands. From another perspective, due to the primal evolutionary relationship between fungi and humans, some drugs that have a cytotoxic effect on fungi may be deleterious to humans (151, 152). Therefore, the use of promising, novel techniques and the combination of innovative screening methods with new chemical formulations can significantly advance the field of personalized drug discovery to counteract fungal and invasive *Candida* infection and subsequently hinder cancer progression. 

ADH: Alcohol dehydrogenases

## Conclusion

Innovation in drug development is highly demanded to control and eliminate candidiasis in cancer patients and consequently prevent cancer progression. However, a simultaneous anti-tumor effect should be another property of this concern.
